# Physical therapy modalities for treating fibromyalgia

**DOI:** 10.12688/f1000research.17176.1

**Published:** 2019-11-29

**Authors:** Fernanda Mendonça Araújo, Josimari Melo DeSantana

**Affiliations:** 1 Graduate Program in Physiological Science, Federal University of Sergipe, Cidade Universitária Prof. José Aloísio de Campos, Avenida Marechal Rondon, s/n - Jardim Rosa Elze, São Cristóvão, SE, 49100-000, Brazil; 2 Department of Physical Therapy, Federal University of Sergipe, Cidade Universitária Prof. José Aloísio de Campos, Avenida Marechal Rondon, s/n - Jardim Rosa Elze, São Cristóvão, SE, 49100-000, Brazil

**Keywords:** Fibromyalgia, pain, function, physical therapy modalities.

## Abstract

Fibromyalgia is a syndrome characterized by generalized chronic musculoskeletal pain, hyperalgesia in specific points, and psychosomatic symptoms, such as fatigue, sleep disturbances (waking unrefreshed), anxiety, depression, cognitive dysfunction, headache, and gastrointestinal disorders. Investigations with non-pharmacological therapies, focused on physical therapy, have increased in recent years as alternative therapies for the treatment of fibromyalgia. The purpose of this review is to summarize the main physical therapy modalities used to treat fibromyalgia.

Fibromyalgia (FM) is a syndrome that affects millions of people around the world (between 0.2 and 6.6% of the world population), especially women, after 50 years of age ^1– 3^. Despite years of investigation in FM, no definite cause or pathophysiology has yet been discovered. In regard to the symptomatology, it is known to be a syndrome characterized by generalized chronic musculoskeletal pain (more than 3 months’ duration) with hyperalgesia in specific points, called “tender points” ^4^.

In addition, patients with FM have associated psychosomatic symptoms, such as fatigue, sleep disturbances (waking unrefreshed), anxiety, depression, cognitive dysfunction, headache, and gastrointestinal disorders ^4, 5^. Thus, clinical presentation of FM usually occurs with the reduction of functional capacity and, consequently, reduction of patients’ quality of life ^6– 8^.

In the face of the variety of symptoms, FM is complex and heterogeneous, and, to date, there is no definitive test to confirm its diagnosis. Therefore, its diagnosis and treatment are very complex. FM has no definitive cure, and treatment focuses primarily on managing symptoms and improving quality of life. This treatment strategy entails a comprehensive multidisciplinary approach that consists of pharmacologic measures, lifestyle modifications, and other complementary approaches. Pharmacological therapy is one of the most indicated but this generates some side effects, besides having a low level of recommendation ^9^. Investigations with non-pharmacological therapies, focused on physical therapy, have increased in recent years as alternative therapies with fewer or no side effects to patients.

Thus, the purpose of this review is to summarize the main physical therapy modalities used to treat FM. These modalities, which will be addressed separately, are aerobic exercises, resistance exercises for muscle strengthening, stretching exercises, aquatic exercises, massage therapy, and therapies with electrical analgesic currents.

Physical exercise, the most strongly indicated non-pharmacological therapy, is based on aerobic exercises, resistance exercise for muscle strengthening, and stretching exercises ^9^. A recent systematic review collected evidence from 13 studies that investigated the effect of aerobic exercise on pain intensity, quality of life, stiffness, fatigue, and physical function of patients with FM ^10^. In that review, the authors concluded that aerobic exercise presented moderate evidence to improve quality of life (evaluated through the Fibromyalgia Impact Questionnaire, or FIQ). There was a low level of evidence for the improvement of pain intensity and physical function. There were minimal differences regarding fatigue and stiffness.

However, few articles assessed fatigue (three studies) and rigidity (one study) as part of their outcome measures, and it was observed that there is great heterogeneity between the described aerobic exercise protocols. These facts may compromise the results presented by the review, besides making it difficult to choose better parameters for treatment efficacy.

Articles included in the review had exercise protocols that had varied frequency (two or three times a week), a mean duration of 35 minutes, and low to moderate intensity (dosed by maximal heart rate ranging from 60 to 75%) and that lasted for 6 to 24 weeks ^10^. Type of aerobic exercises—including protocols with walking, running, cycling, dance, and water exercises—also diverged. The review did not point out which was the best protocol to use. Therefore, the results should be analyzed with caution, and further studies are recommended to verify the effectiveness of aerobic exercise.

Another recommended modality of exercise is muscle strengthening exercise. Individuals with FM have reduced muscle strength, which contributes to a reduction of functionality previously mentioned ^6^. In a Cochrane Review, resistance exercise had a low level of evidence for improvement of multidimensional function (assessed by FIQ), physical function, pain intensity, muscle strength, and sensitivity (tender point count) ^11^. Only five studies were included, and one to three included studies were used for a meta-analysis in which the variables were investigated. As with aerobic exercise, treatment protocols diverged between studies; the exercise was performed two or three times a week, from 30 to 90 minutes, for 16 to 21 weeks. In addition, the intensity of the exercise varied practically in all five included studies, which involved free weights, 40 to 60% of a maximal repetition (1 repetition maximum), 4 out of 10 points on the Borg scale, weights of 1 to 3 pounds (0.45 to 1.36 kg), and elastic bands ^11^.

Flexibility exercises are indicated for the purposes of relieving muscle tension and increasing muscle length and consequently range of motion. Only four articles were included in a previous systematic review ^12^. These studies concluded that flexibility exercises are effective for reducing pain, FM impact, fatigue, and sleep disorders and for improving quality of life and muscle flexibility. However, included studies presented moderate methodological quality (mean of 5 points on the PEDro scale) and this exercise modality did not show superiority to muscle strengthening exercise or laser therapy. In addition, owing to the lack of studies and the heterogeneity of the included studies, it was not possible to perform a meta-analysis. There was also a lack of detailed description of the flexibility exercise protocols used ^12^.

Besides land exercises, water exercises have been recommended for treatment of FM ^9, 13^. Hydrodynamic properties of water, such as buoyancy, density, viscosity, and hydrostatic pressure, provide resistance to movement, leading to muscle strengthening and causing muscle relaxation, low joint impact, and better venous return ^14^.

In individuals with FM, aquatic therapy showed moderate to strong evidence for small pain reduction and small improvement in quality of life ^15^. No significant effect was found for depressive symptoms and tender point count. The treatment protocol ranged from 5 weeks to 8 months, had a frequency of one to three times a week (in one study, the treatment occurred daily), and a duration of 30 to 60 minutes. When reported, the water temperature was set at 28°C to 34 °C.

Physical therapy modalities that passively treat FM have also been investigated. In a previous systematic review, six types of massage therapy (Swedish massage, connective tissue massage, manual lymphatic drainage, myofascial release, Shiatsu, and a combination of severe massage styles) applied one to five times a week, for 4 to 40 weeks of treatment, were investigated as a therapeutic resource for patients with FM ^16^. Myofascial release has been shown to be more effective than placebo for improving pain, fatigue, stiffness, anxiety, depression, and quality of life of individuals with FM (moderate level of evidence) ^16^. Eight of the ten included articles in the review compared different types of massage therapy among themselves (with no placebo group) and showed limited scientific evidence for effectiveness of the other types of massage therapy. In addition, Swedish massage appears to be contraindicated because of the lack of benefit to patients ^16^.

Finally, therapies with electrical analgesic currents have been used in the treatment of FM. A recent literature review distinguishes two types of electrostimulation: non-invasive (transcutaneous electrical nerve stimulation, or TENS, whose application occurs by electrodes on the skin) and invasive (electroacupuncture, which is the combination of current and acupuncture) ^17^. It was concluded that there is a low quality of evidence that TENS is effective for relieving pain in patients with FM. Electroacupuncture presented a moderate level of evidence for pain relief. There was no effect on the quality of life and fatigue of these patients ^17^. However, only nine articles were included: four with TENS, two with TENS associated with physical exercise, and three with electroacupuncture. In addition to including a low number of studies, they presented a high risk of bias, and there was no concealment of subjects’ allocation, no blinding of patient and investigators, and no intention-to-treat analysis.

In the treatment protocol, the currents were applied for at least 20 to 40 minutes with different stimulation frequencies (4, 80, 100, and 150 Hz) ^17^. Despite the importance of the stimulation intensity for the production of hypoalgesia ^18^, the stimulation intensities used in the included studies were not mentioned. In addition, studies investigating the use of interferential current (IFC) in patients with FM were not included. IFC is an electrical current of medium frequency, applied to the skin, to promote pain relief ^19, 20^. A recent systematic review showed that this current is an effective therapy for reducing pain and improving sleep quality in patients with FM ^21^. However, only one article was included in the review, in which IFC was not applied as a single strategy, but in combination. Thus, it is not possible at this time to verify the real effectiveness of IFC in patients with FM.

In summary, articles about the effectiveness of non-pharmacological (physical therapy modalities) resources in individuals with FM show considerable variability, varied treatment protocols, and methodological quality problems ^22^. These facts have led to low evidence quality and hampered the process of choosing better treatment protocols. According to Mannerkorpi and Iversen (2003), individuals with FM are part of a heterogeneous group and therefore exercise interventions must be individualized on the basis of the patient’s physical function, severity of pain, and other FM symptoms ^23^.

Despite the diversity of symptoms and heterogeneity present in individuals with FM, few outcomes were investigated in the studies, which resulted in the small number of articles included in the meta-analyses. Pain intensity, fatigue, anxiety, depression, and quality of life should always be investigated. Despite limitations in the design of these studies, physical therapy seems to improve symptoms present in patients with FM and to have no side effects. However, more studies with adequate methodological quality and standardized treatment protocols are necessary to better investigate the effectiveness of these physical therapy modalities in the various characteristic symptoms of individuals with FM.

## Abbreviations

FIQ, Fibromyalgia Impact Questionnaire; FM, fibromyalgia; IFC, interferential current; TENS, transcutaneous electrical nerve stimulation

## References

[1] Cabo-Meseguer A Cerdá-Olmedo G Trillo-Mata JL: Fibromyalgia: Prevalence, epidemiologic profiles and economic costs. *Med Clin (Barc).* 2017;149(10):441–8. 10.1016/j.medcli.2017.06.008 28734619

[2] Marques AP Santo ASDE Berssaneti AA: Prevalence of fibromyalgia: literature review update. *Rev Bras Reumatol Engl Ed.* 2017;57(4):356–63. 10.1016/j.rbre.2017.01.005 28743363

[3] Heidari F Afshari M Moosazadeh M: Prevalence of fibromyalgia in general population and patients, a systematic review and meta-analysis. *Rheumatol Int.* 2017;37(9):1527–39. 10.1007/s00296-017-3725-2 28447207

[4] Wolfe F Smythe HA Yunus MB: The American College of Rheumatology 1990 Criteria for the Classification of Fibromyalgia. Report of the Multicenter Criteria Committee. *Arthritis Rheum.* 1990;33(2):160–72. 10.1002/art.1780330203 2306288

[5] Wolfe F Walitt B Perrot S: Fibromyalgia diagnosis and biased assessment: Sex, prevalence and bias. *PLoS One.* 2018;13(9):e0203755. 10.1371/journal.pone.0203755 30212526 PMC6136749

[6] Góes SM Leite N Shay BL: Functional capacity, muscle strength and falls in women with fibromyalgia. *Clin Biomech (Bristol, Avon).* 2012;27(6):578–83. 10.1016/j.clinbiomech.2011.12.009 22230426

[7] Lee JW Lee KE Park DJ: Determinants of quality of life in patients with fibromyalgia: A structural equation modeling approach. *PLoS One.* 2017;12(2):e0171186. 10.1371/journal.pone.0171186 28158289 PMC5291376

[8] Álvarez-Gallardo IC Soriano-Maldonado A Segura-Jiménez V: High Levels of Physical Fitness Are Associated With Better Health-Related Quality of Life in Women With Fibromyalgia: The al-Ándalus Project. *Phys Ther.* 2019;pii: pzz113. 10.1093/ptj/pzz113 31392995

[9] Macfarlane GJ Kronisch C Dean LE: EULAR revised recommendations for the management of fibromyalgia. *Ann Rheum Dis.* 2017;76(2):318–28. 10.1136/annrheumdis-2016-209724 27377815

[10] Bidonde J Busch AJ Schachter CL: Aerobic exercise training for adults with fibromyalgia. *Cochrane Database Syst Rev.* 2017;6:CD012700. 10.1002/14651858.CD012700 28636204 PMC6481524

[11] Busch AJ Webber SC Richards RS: Resistance exercise training for fibromyalgia. *Cochrane Database Syst Rev.* 2013;28(12):CD010884. 10.1002/14651858.CD010884 24362925 PMC6544808

[12] Lorena SB Lima Mdo C Ranzolin A: Effects of muscle stretching exercises in the treatment of fibromyalgia: A systematic review. *Rev Bras Reumatol.* 2015;55(2):167–73. 10.1016/j.rbr.2014.08.015 25440706

[13] Bidonde J Busch AJ Webber SC: Aquatic exercise training for fibromyalgia. *Cochrane Database Syst Rev.* 2014; (10):CD011336. 10.1002/14651858.CD011336 25350761 PMC10638613

[14] Torres-Ronda L Del Alcázar XS: The Properties of Water and their Applications for Training. *J Hum Kinet.* 2014;44:237–48. 10.2478/hukin-2014-0129 25713684 PMC4327375

[15] Naumann J Sadaghiani C: Therapeutic benefit of balneotherapy and hydrotherapy in the management of fibromyalgia syndrome: A qualitative systematic review and meta-analysis of randomized controlled trials. *Arthritis Res Ther.* 2014;16(4):R141. 10.1186/ar4603 25000940 PMC4227103

[16] Yuan SL Matsutani LA Marques AP: Effectiveness of different styles of massage therapy in fibromyalgia: A systematic review and meta-analysis. *Man Ther.* 2015;20(2):257–64. 10.1016/j.math.2014.09.003 25457196

[17] Salazar AP Stein C Marchese RR: Electric Stimulation for Pain Relief in Patients with Fibromyalgia: A Systematic Review and Meta-analysis of Randomized Controlled Trials. *Pain Physician.* 2017;20(2):15–25. 28158150

[18] Vance CG Dailey DL Rakel BA: Using TENS for pain control: The state of the evidence. *Pain Manag.* 2014;4(3):197–209. 10.2217/pmt.14.13 24953072 PMC4186747

[19] Nemec H: Interferential therapy: a new approach in physical medicine. *Br J Physiother.* 1959;12:9–12.

[20] Almeida CC Silva VZMD Júnior GC: Transcutaneous electrical nerve stimulation and interferential current demonstrate similar effects in relieving acute and chronic pain: A systematic review with meta-analysis. *Braz J Phys Ther.* 2018;22(5):347–54. 10.1016/j.bjpt.2017.12.005 29426587 PMC6157468

[21] Silva MTd Araújo FM Araújo MF: Effect of interferential current in patients with fibromyalgia: A systematic review. *Fisioter. Pesqui.* 2018;25(1):107–14. 10.1590/1809-2950/17276725012018

[22] Álvarez-Gallardo IC Bidonde J Busch A: Therapeutic validity of exercise interventions in the management of fibromyalgia. *J Sports Med Phys Fitness.* 2019;59(5):828–38. 10.23736/S0022-4707.18.08897-7 30293405

[23] Mannerkorpi K Iversen MD: Physical exercise in fibromyalgia and related syndromes. *Best Pract Res Clin Rheumatol.* 2003;17(4):629–47. 10.1016/s1521-6942(03)00038-x 12849716

